# Kinetics, Electronic Properties of Filled Carbon Nanotubes Investigated with Spectroscopy for Applications

**DOI:** 10.3390/nano13010176

**Published:** 2022-12-30

**Authors:** Marianna V. Kharlamova

**Affiliations:** Centre for Advanced Materials Application (CEMEA), Slovak Academy of Sciences, Dúbravská cesta 5807/9, 845 11 Bratislava, Slovakia; dr.marianna.kharlamova@gmail.com

**Keywords:** carbon nanotube, spectroscopy, growth kinetics, electronic properties, Raman spectroscopy, near edge X-ray absorption fine-structure spectroscopy, photoemission spectroscopy, optical absorption spectroscopy

## Abstract

The paper is dedicated to the discussion of kinetics of growth, and electronic properties of filled carbon nanotubes investigated by spectroscopy for applications. The paper starts with discussion of growth of carbon nanotubes inside metallocene-filled carbon nanotubes. Nickelocene, cobaltocene are considered for growth of carbon nanotubes. Then, the investigations of filled carbon nanotubes by four spectroscopic techniques are discussed. Among them are Raman spectroscopy, near edge X-ray absorption fine-structure spectroscopy, photoemission spectroscopy, optical absorption spectroscopy. It is discussed that metal halogenides, metal chalcogenides, metals lead to changes in electronic structure of nanotubes with n- or p-doping. The filling of carbon nanotubes with different organic and inorganic substances results in many promising applications. This review adds significant contribution to understanding of the kinetics and electronic properties of filled SWCNTs with considering new results of recent investigations. Challenges in various fields are analyzed and summarized, which shows the author’s viewpoint of progress in the spectroscopy of filled SWCNTs. This is a valuable step toward applications of filled SWCNTs and transfer of existing ideas from lab to industrial scale.

## 1. Introduction

Single-walled carbon nanotubes (SWCNTs) have attracted the attention of researchers, because they can be filled with more than 190 different substances, such as metal halogenides, metal chalcogenides, metals, nonmetals, and molecules. Several groups work on filled carbon nanotubes in various countries across Europe, including Austria. Besides investigations of different molecules [1,2,3,4,5,6,7,8,9,10,11,12,13,14,15,16,17,18,19], for the modification of the electronic properties, the SWCNTs were filled with halogenides of 3d-metal MX_2_ (M = Fe, Co, Ni, Mn, Zn, Cu, X = Cl, Br, I) [20,21,22,23,24,25,26,27,28,29,30,31,32,33], 4d-metal MX_2_ (M = Cd, Ag, X = Cl, Br, I) [34,35,36,37,38,39], 5d-metals MX_2_ (M = Hg, X = Cl) [40] and 4f-metal MX_3_ (M = Pr, Tb, Tm, Lu, X = Cl, Br, I) [41,42,43,44,45], 6p-metal MX_2_ (M = Pb, X = Cl, Br, I) [46], 5s-metal MX (M = Rb, X = I) [47], 6s-metal MX (M = Cs, X = I) [48], metal chalcogenides A^III^B^VI^ (GaX, X = Se, Te) [49,50], A^IV^B^VI^ (SnX, X = S, Se, Te) [51] and A^V^B^VI^ (Bi_2_X_3_, X = Se, Te) [50], metals (Ag, Cu, Eu, Cs) [52,53,54,55,56,57], nonmetals (I_2_, S, Se, Te) [52,57,58,59], organic [60] and organometallic compounds—metallocenes (C_5_H_5_)_n_M (M = Fe, Co, Ni, Ce, n = 2, 3) [61,62,63,64,65,66,67,68,69,70,71,72,73,74,75,76] and metal acetylacetonates M(C_5_H_7_O_2_)_2_ (M = Ni, Pt) [77,78]. 

The growth properties of nanotubes inside filled nanotubes are studied by Raman spectroscopy. It is found that substances formed upon thermal treatment of metallocene-filled nanotubes catalyze the synthesis of inner nanotubes. These substances are metals and metal carbides. The inner tubes are formed within several minutes to several days, depending on synthesis temperature [67,70]. 

The electronic properties of filled and annealed samples are studied with four methods, Raman spectroscopy, near edge X-ray absorption fine-structure spectroscopy, photoemission spectroscopy, and optical absorption spectroscopy for applications of SWCNTs [79,80,81,82]. 

## 2. New Era of Filled Carbon Nanotubes Is Coming

Solar cells with specialized properties are among the envisaged application of filled SWCNTs. Silicon solar cells with carbon nanotube top electrodes are functional but lack in efficiency and stability [83]. The photovoltaic performance of SWCNT-silicon solar cells is, however, tuneable by tailoring the SWCNT films via chemical doping, improving the SWCNT-silicon and SWCNT–metal interfaces and the use of antireflection layers [83]. To date, the silicon solar cells with SWCNTs top electrodes have been tested with exohedrally doped SWCNTs [84,85,86,87,88,89,90,91,92,93]. While exohedral doping is technologically simpler and does improves the device performance, the stability over extended periods of time as well as chemical inertness and mechanical robustness cannot be addressed by the exohedral approach. We want to suggest that these challenges could be overcome by applying filled and endohedrally doped SWCNT for top electrodes in solar cells [84,85,86,87,88,89,90,91,92,93]. There are, however, to the best of our knowledge, no reports on the use of the filled SWCNTs in nanotube-silicon solar cells so far. The stable and uniform doping of SWCNTs achievable by filling SWCNTs with carefully selected dopants would allow improving the power conversion efficiency (PCE) of solar cells while simplifying the fabrication process and its scalability. 

Perovskite solar cells (PSCs) with top metal electrodes require a costly production process where gold or silver layers are deposited under high vacuum [94]. The very thin metal electrodes are found to be very fragile, which limits the long-term stability in ambient conditions [94]. In the PSCs, films of SWCNTs are already outperforming the conventional fragile metal electrodes since the SWCNT films feature stability, abundant availability, mechanical robustness and hydrophopic properties [95].

Replacing the top metal electrodes of PSCs with SWCNT films substantially enhances the stability of PSCs by cancelling the otherwise unavoidable metal ion migration and drastically simplifies the fabrication process as they can be deposited with a simple mechanical transfer [94]. Furthermore, shifting the Fermi level in the SWCNT electrodes allows enhancing the PCE of the SWCNT electrode-based PSCs [95].

To date, PSCs with top electrodes were made from exohedrally doped SWCNTs [95,96,97,98,99]. We suggest that the use of filled SWCNTs with a tailored Fermi level position will allow achieving stable PSCs with increased photovoltaic performance. 

Organic solar cells (OSCs) are envisaged as a green low cost technology that can be surface conforming and flexible and thus wearable. For such applications, there is a clear need for metal-free, mechanically resilient, and translucent materials that can support a high PCE. A way to fulfill these requirements is to substitute the top metal electrode, which is fragile, expensive, and very reflective, by transparent conductive SWCNT electrodes [83]. 

Such transparent and flexible organic solar cells with high PCEs have been demonstrated with films of SWCNTs through exohedral doping [96,100,101]. The implementation of filled SWCNTs in OSCs has not yet been demonstrated; however, the improved stability and efficiency achievable by encapsulating the dopant inside the SWCNTs are bound to be advantages in various applications. 

Filled SWCNTs are in the form of transparent thin films an interesting material for the top electrodes in solar cells. They offer solutions to the current challenges in the technology. They can at once benefit the power conversion efficiency, improve the chemical and mechanical robustness and durability, and increase thermal and operational stability. Very importantly, they can simplify the production process by skipping steps in ultra-high vacuum and reduce the production costs.

The promising application of filled SWCNTs is based on the thermoelectric power generation effect in filled SWCNTs. The connecting of parts that are p-doped and n-doped leads to the creation of the effect in the device. Thermoelectric applications are aiming at the highest possible conversion efficiency, which can be achieved by an increased electrical conductivity and a decreased thermal conductivity. 

SWCNTs with patterned filling and patterned modified electronic properties would be ideal for application in light-emitting devices. To date, SWCNT-based light-emitting p-n diodes were realized by electrostatic and exohedral doping [102,103]. Undoubtedly, overcoming the challenges in precisely controlled patterning in filled SWCNTs will lead to the next generation of SWCNT-based light-emitting devices with further miniaturization and improved stability and longevity. 

If filled SWCNT are used as light-emitting p-n-diodes, they operate with a high carrier-to-photon emission efficiency without any threshold current. This does not only increase power efficiency but also reduces power dissipation and unintended heating. This will allow to simplify designs and also cut down operational costs of light emitting devices. 

The promising application of filled SWCNTs is in devices with piezoelectric effect.

New devices of the future can be created by combining the above-mentioned devices. They open new era in science, technology, health care and military equipment. 

## 3. Filling Methods of SWCNTs

There are three methods of filling of SWCNTs: gas phase, liquid solution, and melt methods.

In the gas phase approach, the SWCNTs are filled with gaseous substances. The filling upon heating takes place inside a quartz ampoule where the substance vaporizes and diffuses. Upon cooling, the substance crystallizes [23,24]. This is a simple method, however, the melting point of the substance should not be too high (below 1200 °C), because the nanotubes can decompose. 

In the solution approach, the SWCNTs are filled with solution of substance. The filling takes place inside the glass flask where the solution goes inside the carbon nanotubes. After that, the multi-step procedures of cleaning take place [23,24]. Then, the SWCNTs filled with solution undergo the thermal treatment to chemically transform to new substance. 

In the melt approach, the SWCNTs are filled with melt of substance. The filling takes place in a quartz ampoule where the melt incorporates inside the carbon nanotubes. After that, the cooling of system is made [23,24]. Then, the cleaning procedures of filled carbon nanotubes are performed. The substances can have high melting point. The filling ratio that is achieved is large. 

## 4. Remarks on Characterization Methods of Filled SWCNTs

Raman spectroscopy is nondestructive method of investigation of the electronic properties of SWCNTs. The shifts of bands and their modifications are analyzed, and modification of the electronic properties is studied [25,26,27,28,29,30,31,32,33,34,35,36,37,38,39,41,42,43,44,46,47,49,50,51,55,56,57,61,62,63,64,65,67,68,69,70,71,72,73,74]. 

Raman spectroscopy is a simple method that includes the irradiation of a sample with laser beam, and measuring the spectrum. The spectrum is the dependence of intensity of the scattered light on Raman shift [104]. The latter is the difference in energy between initial and scattered phonons. The Raman spectroscopy reveals different types of bonds in the analyzed SWCNTs. 

The near edge X-ray absorption fine-structure spectroscopy (NEXAFS) investigates X-ray absorption band near the band edge. It shows a fine structure, which is a signature of this material. Additional peaks that appear in the spectra are investigated [25,26,27,30,32,34,36,41,49,51]. 

The X-ray absorption spectroscopy (XAS) studies local geometric and electronic structure. The sample is irradiated with synhrotron light, adsorbs by the sample, and it originates series of absorption bands [104]. The intensity of signal is very high. The XAS reveals different elements, because the signal is characteristic to different chemical elements. 

The photoemission spectroscopy investigates the electronic properties of material. The X-ray photoelectron spectroscopy (XPS) [25,26,27,28,39,40,41,42,44,46,47,49,50,51,55,56,57,61,63,64,68,69,72] and ultraviolet photoelectron spectroscopy (UPS) study modifications of positions, width and shape of spectra [30,32,64]. 

Photoemission spectroscopy is an informative method that includes the irradiation of a sample with X-ray, ultraviolet irradiation, and measuring the spectrum [104]. The spectrum is the dependence of kinetic energy of photoelectrons on binding energy. In XPS, the photoelectrons are ejected from the core levels, and in UPS, they are ejected from the shallow valence band levels This gives the information on the chemical environment.

The optical absorption spectroscopy studies changes in the optical transitions of the material, including the modifications of the positions of peaks, and their width assigned to the alteration of the electronic properties of SWCNTs [25,26,27,30,31,32,34,36,41,49,51,55,58]. 

Optical absorption spectroscopy is a simple method that includes the irradiation of SWCNTs with the light, and measuring the spectrum [104]. The spectrum is the dependence of the absorbance (or transmittance) on the wavelength. The spectrum gives information about concentrations of SWCNTs, electronic and vibrational transitions. The method is used to investigate the synthesis, properties, and applications of material.

## 5. Literature Review

It is found that different fillers lead to changes in electronic structure of nanotubes with n- or p-doping. The insertion of metal halogenides, metal chalcogenides, metallocenes, and metals leads to the Fermi level shift of −0.3–0.4 eV, −0.3 eV, +0.05–0.1 eV, and +0.3 eV [25,26,27,28,29,30,31,32,33,34,35,36,37,38,39,41,42,43,44,46,47,49,50,51,55,56,57,61,62,63,64,65,67,68,69,70,71,72,73,74]. The encapsulation of rubidium iodide leads to the Fermi level shift of +0.2 eV, i.e., donor doping [47]. There are nine cases of the electronic structure of SWCNTs:

- Pristine SWCNTs where the Fermi level is positioned in the middle between the conduction band and valence band;
- Metal-halogenide-filled SWCNTs where the Fermi level is shifted to conduction band, because the work function of SWCNTs is lower than the work function of SWCNTs; this is the case without the formation of chemical bonds between the SWCNTs and introduced salts;
- Metal-halogenide-filled SWCNTs where the Fermi level is shifted to conduction band, because the work function of SWCNTs is lower than the work function of SWCNTs; this is the case with the formation of chemical bonds between the SWCNTs and introduced salts;
- Metal-halogenide-filled SWCNTs where the Fermi level is shifted to valence band, because the work function of SWCNTs is larger than the work function of salt,
- Metal-chalcogenide-filled SWCNTs where the Fermi level is shifted to conduction band, because the work function of SWCNTs is lower than the work function of SWCNTs;
- Metal-chalcogenide-filled SWCNTs where the Fermi level is not shifted upon filling, because the work functions of SWCNTs and compounds are similar;
- Metal-filled SWCNTs where the Fermi level is shifted to valence band, because the work function of SWCNTs is larger than the work function of metal;
- Molecule-filled SWCNTs where the Fermi level is shifted to conduction band, because the work function of SWCNTs is lower than the work function of molecules;
- Molecule-filled SWCNTs where the Fermi level is shifted to valence band, because the work function of SWCNTs is larger than the work function of molecules.

These precise Fermi level shifts are interesting for the research community and lead to many applications, which can be realized now in different laboratories toward industry scale [82,83,84,85,86,87,88,89,90,91,92,93,94,95,96,97,98,99,100,101,102,103]. As an example, we consider here the data of cobalt diiodide and nickel metal. The aim of this work is the investigation of the electronic properties of filled SWCNTs and revealing the effect of fillers on nanotubes. It was shown that the introduced substances can lead to p- or n-doping of SWCNTs with lowering or increasing the Fermi level of SWCNTs, accordingly. 

## 6. Kinetics of Nanotube Growth

**Figure 1:**
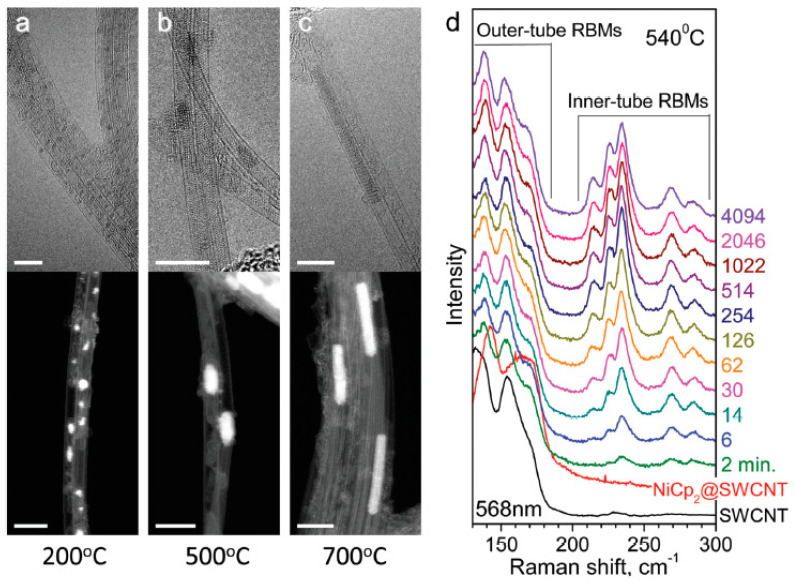
The high-resolution transmission electron microscopy data of the nickelocene-filled SWCNTs annealed at 200 (**a**), 500 (**b**) and 700 °C (**c**). Scale bar, 5 nm. (**d**) The Raman spectroscopy data of the pristine, nickelocene-filled SWCNTs and samples annealed at 540 °C during 2-4094 min measured at laser wavelengths of 568 nm. Reproduced from Ref. [67] with permission from the Royal Society of Chemistry. This article is licensed under a Creative Commons Attribution 3.0 Unported License.

Figure 1 shows the high-resolution transmission electron microscopy (HR TEM) data of the nickelocene-filled SWCNTs annealed at 200, 500, and 700 °C [67]. These data present clusters of nickel with different length, which are formed during annealing. The lengths of typical clusters were estimated to be 3–5 and 10–20 nm at 500 and 700 °C, respectively.

Figure 1d shows the Raman spectroscopy results of the pristine, nickelocene-filled SWCNTs and samples annealed at 540 °C during 2–4094 min measured at 568 nm-laser [67]. The peaks of inner tubes appear, and their intensities grow during annealing, which is accompanied by the formation of long nickel clusters. The small diameter tubes grow faster than the large diameter tubes.

**Figure 2:**
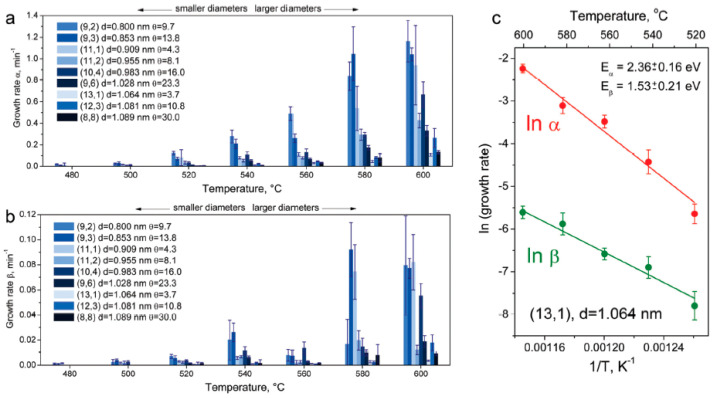
The column bar diagrams summarizing evaluated growth rates α (**a**) and β (**b**) of the (8,8), (12,3), (13,1), (9,6), (10,4), (11,2), (11,1), (9,3), and (9,2) inner tubes at different annealing temperatures. (**c**) Logarithmic growth rates α and β versus inverse annealing temperature for the (13,1) inner tube. *d* is diameter, θ is chiral angle. E_α_ and E_β_ are activation energies. Reproduced from Ref. [67] with permission from the Royal Society of Chemistry. This article is licensed under a Creative Commons Attribution 3.0 Unported License.

The growth rates of inner nanotubes with chiralities of (8,8), (12,3), (13,1), (9,6), (10,4), (11,2), (11,1), (9,3), (9,2) were calculated (Figure 2a,b). The linear fits of logarithmic plots of growth rates for the (13,1) are plotted in Figure 2c [67]. 

**Figure 3:**
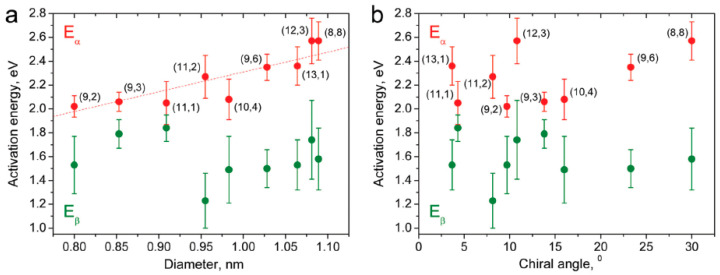
Activation energies Eα and Eβ for the growth of the inner tubes with chiral vectors of (8,8), (12,3), (13,1), (9,6), (10,4), (11,2), (11,1), (9,3), and (9,2) plotted versus the tube diameter (**a**) and chiral angle (**b**). Reproduced from Ref. [67] with permission from the Royal Society of Chemistry. This article is licensed under a Creative Commons Attribution 3.0 Unported License.

Taking into account the growth rates, the activation energy was calculated. In Figure 3 the dependence of activation energy on the tube diameter and chiral angle are presented. Figure 3a demonstrates that E_α_ (2.02–2.57 eV) is higher than E_β_ (1.23–1.84 eV). The fakel-like behavior of dependence on the chiral angle (Figure 3b) is nearly recognized [67]. We will investigate it additionally in different laboratories to study the mechanism of nanotube growth more precisely.

**Figure 4:**
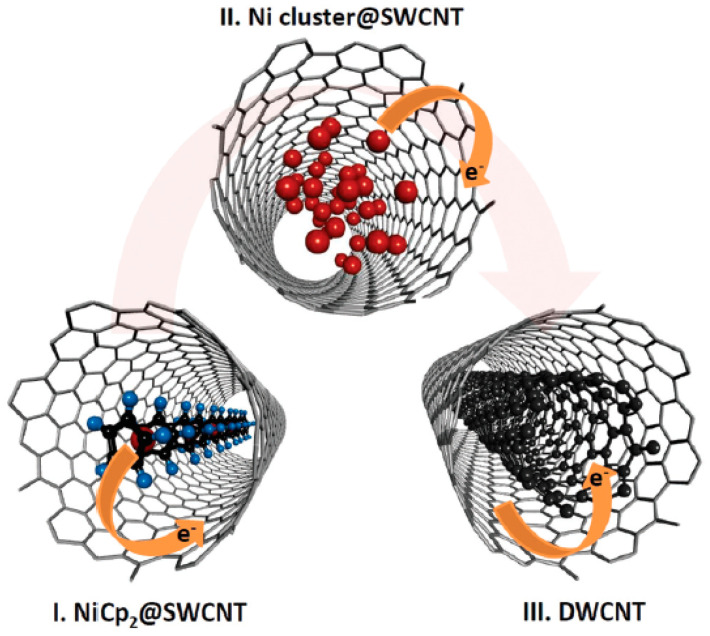
Schematic of electron doping of SWCNTs via the chemical transformation of encapsulated nickelocene. Reproduced from Ref. [64] with permission from the Royal Society of Chemistry. This article is licensed under a Creative Commons Attribution 3.0 Unported License.

The schematics (Figure 4) shows the charge transfer in nickelocene, nickel-filled SWCNTs, and DWCNTs [64]. 

**Figure 5:**
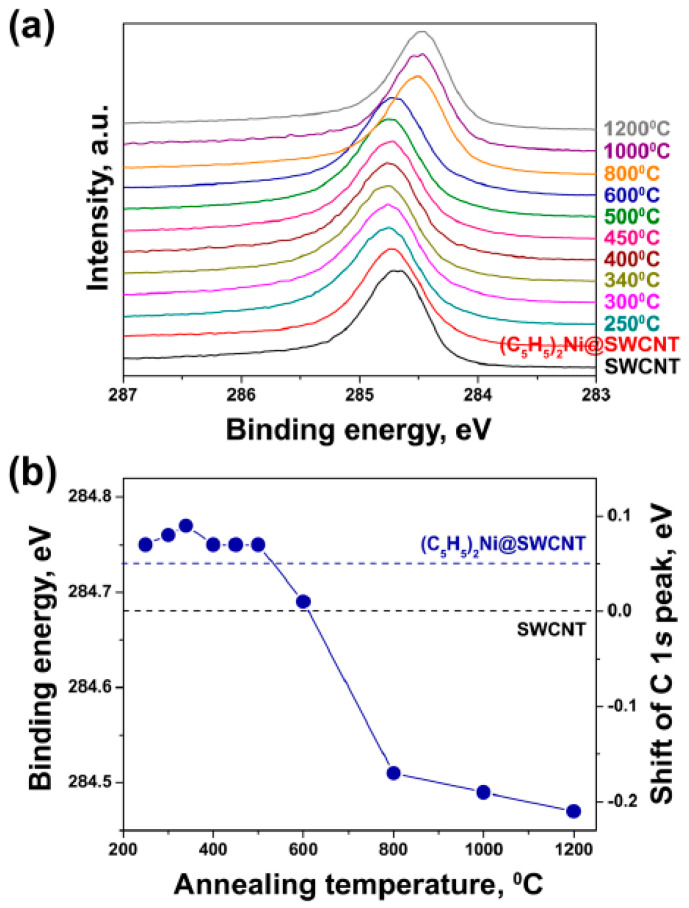
(**a**) The C 1s XPS spectra of nickelocene, nickel-filled SWCNTs and DWCNTs obtained at annealing at temperature from 250 to 1200 °C. (**b**) The C 1s the binding energy and its shift relative to the pristine nanotubes plotted versus the annealing temperature. Reprinted with permission from Ref. [63], copyright 2015 Wiley-VCH Verlag GmbH & Co. KGaA, Weinheim, Germany.

The electronic properties of nickelocene, nickel-filled SWCNTs and DWCNTs obtained at annealing at temperature from 250 to 1200 °C were investigated by XPS. The C 1s XPS spectra (Figure 5a) show the shifts to the high-energy region for annealing at 250 to 600 °C, and to the low-energy region for annealing from 800 to 1200 °C. This testifies to n-doping and p-doping, respectively (Figure 5b) [63]. 

**Figure 6:**
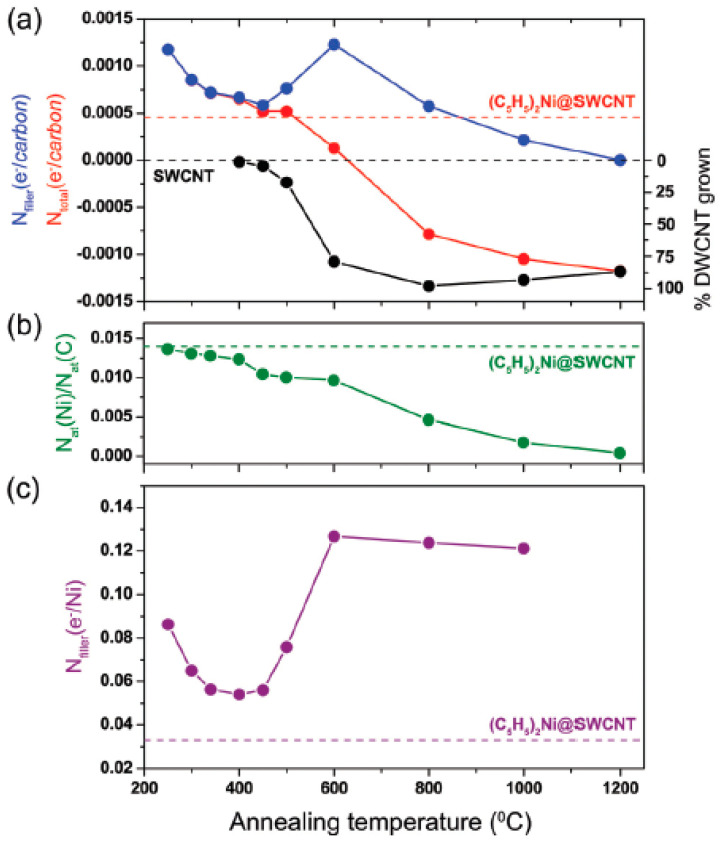
The schematics of method of calculation of number (N) of electrons transferred in nickel-filled SWCNTs per carbon atom (**a**), ratio of number of atoms (**b**), per nickel atom (**c**). Dashed horizontal lines show the values for SWCNTs and nickelocene-filled SWCNTs. Reproduced from Ref. [64] with permission from the Royal Society of Chemistry. This article is licensed under a Creative Commons Attribution 3.0 Unported Licence.

Figure 6 shows the schematics of calculation of charge transfer in nickel-filled SWCNTs [64]. This method led to the calculation of the number of electrons transferred from nickel substances per nickel atom in annealed nickel-filled SWCNTs. 

Thus, kinetics of nickelocene-filled SWCNTs shows that metal-filled SWCNTs catalyze growth of SWCNTs, and it changes the electronic properties of filled SWCNTs.

## 7. Raman Spectroscopy

**Figure 7:**
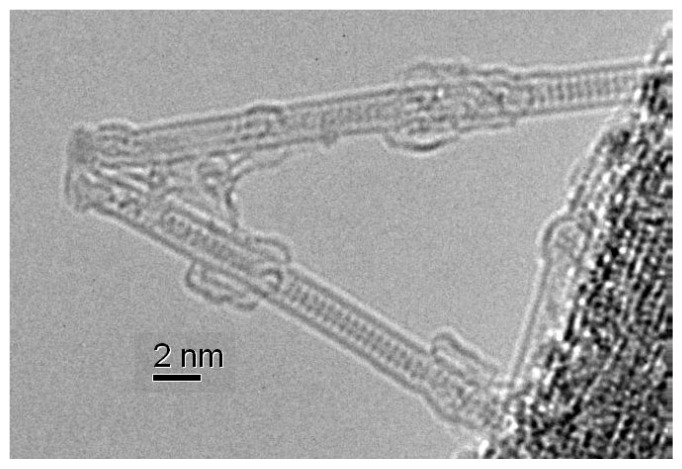
The HR TEM image of cobalt-diiodide-filled SWCNTs [82].

Figure 7 shows the high-resolution transmission electron microscopy HR TEM image of cobalt iodide-filled SWCNTs. The microphoto shows the contrast elements inside walls of carbon nanotubes. They are located in lines of parallel dots inside SWCNTs. This confirms the filling of SWCNTs with salt [82]. 

**Figure 8:**
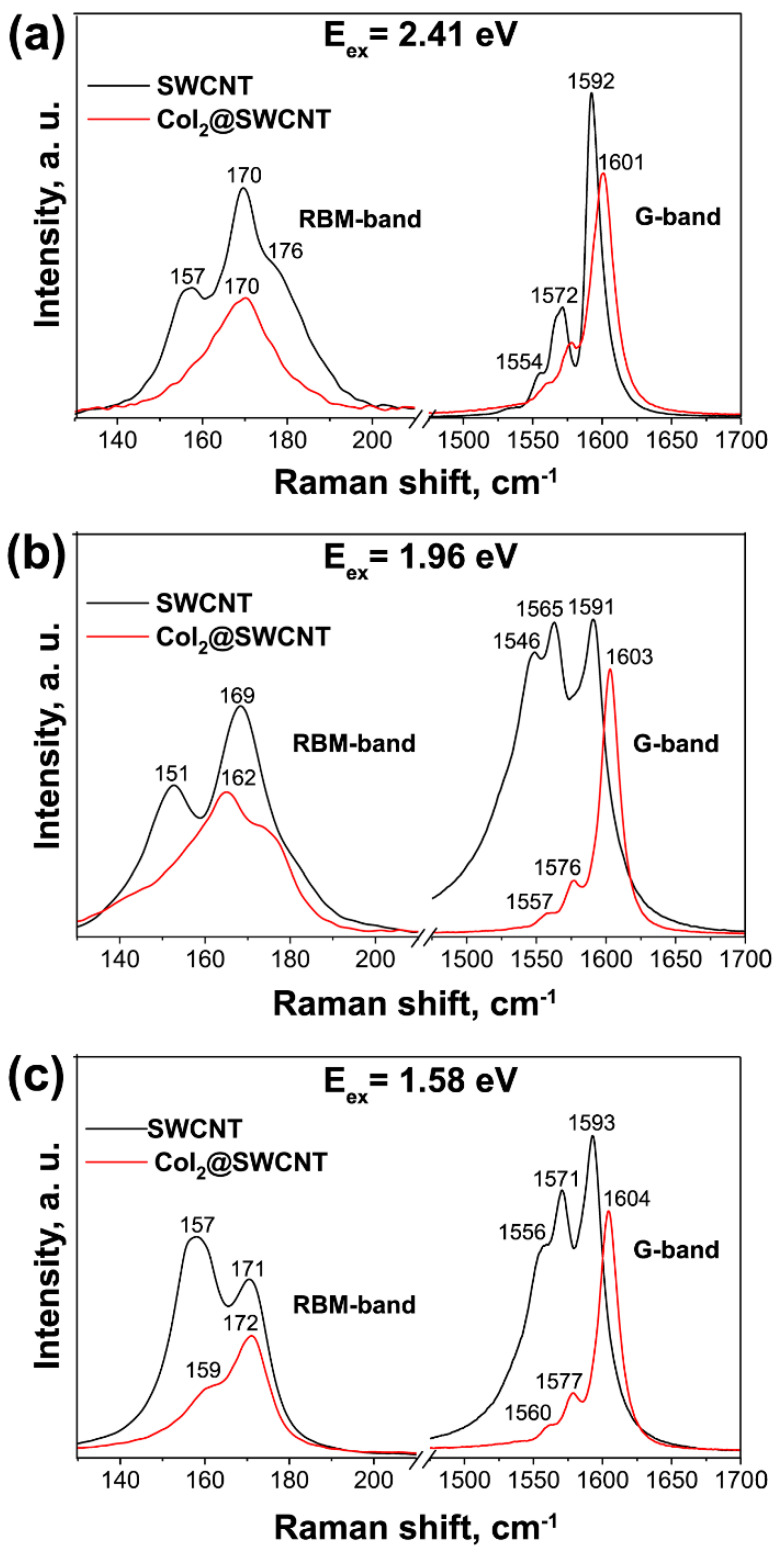
The RBM and G-bands of Raman spectra of pristine and cobalt diiodide-filled SWCNTs acquired at laser wavelengths of 514 (**a**), 633 (**b**) and 785 (**c**) nm. The positions of peaks are marked with numbers.

**Figure 9:**
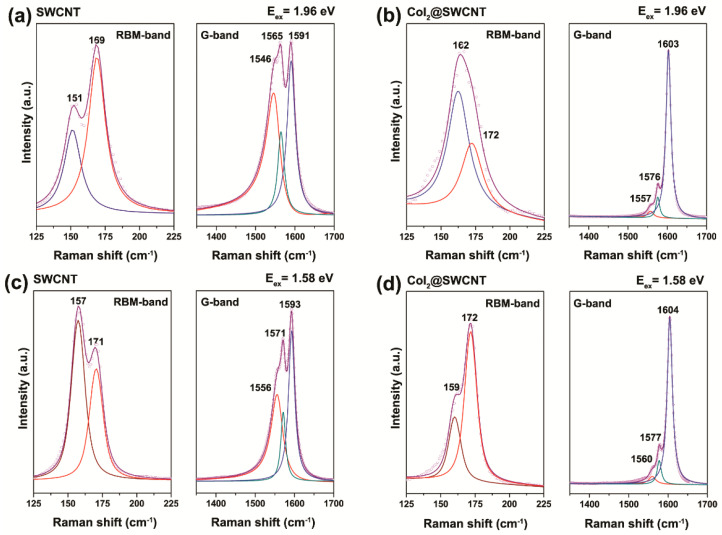
The fitting of RBM and G-band of Raman spectra of the pristine and cobalt-diiodide-filled SWCNTs acquired at laser energies of 1.96 (**a**,**b**, accordingly), and 1.58 eV (**c**,**d**, accordingly). The numbers denote the position of peaks.

Figure 8 shows the Raman spectra of the pristine and cobalt diiodide filled SWCNTs. The radial breathing mode (RBM) and G-band of filled SWCNTs shows the modifications, because the introduced substance leads to alteration of properties of nanotubes. The observed changes are: (i) shift in peaks, (ii) decrease in intensity of peaks, (iii) change in band profile from metallic to semiconducting for metallic excited SWCNTs at laser wavelengths of 633 (Figure 8b) and 785 nm (Figure 8c), (iv) in case of large diameter SWCNTs the disappearance of peaks of RBM-band is observed, (v) the signal from these nanotubes in G-band also disappear. Figure 9 shows the fitting of RBM and G-bands of Raman spectra of pristine and filled SWCNTs (Table 1). The RBM-band is fitted with two components of different-diameter SWCNTs. The G-band is fitted with G_BWF_ (Breit–Wigner–Fano components), G_TO_ tangential phonon component, G_LO_ longitudinal phonon component. 

**Figure 10:**
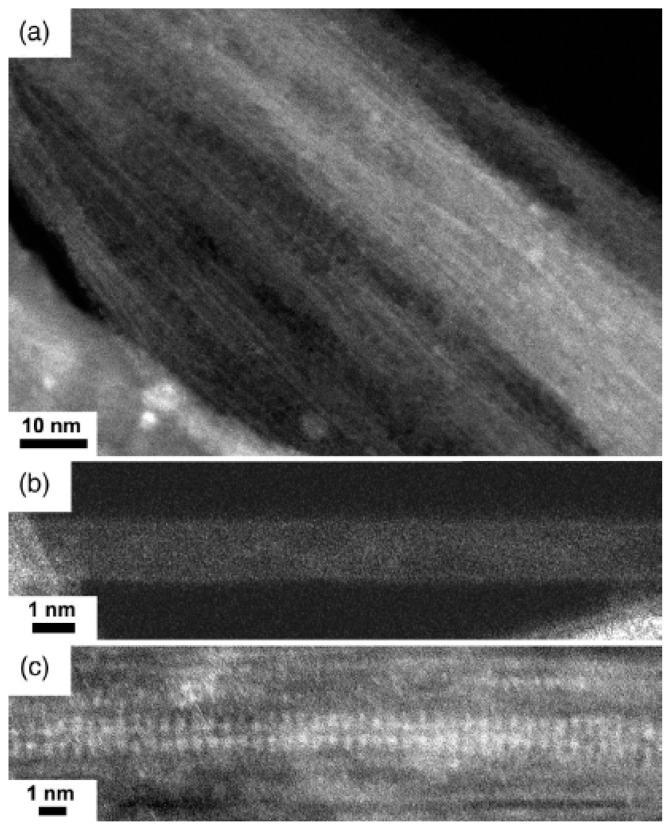
The HRTEM images of rubidium-iodide-filled SWCNTs. (**a**) Low magnification image. (**b**) Empty individual SWCNT. (**c**) Individual filled SWCNT. Reprinted with permission from Ref. [47], copyright 2019 Wiley-VCH Verlag GmbH & Co. KGaA, Weinheim.

Raman spectroscopy studies also the electronic properties of n-doped SWCNTs. Figure 10 shows the TEM images of the rubidium-iodide-filled SWCNTs. The low magnification image of filled SWCNTs shows the filled channels, because the filling process took place (Figure 10a). Figure 10b shows the pristine SWCNT demonstrating the perfect nanotube. Figure 10c presents the filled rubidium iodide containing SWCNTs. Atoms of the compound are visible; paars of contrast lines of white dots are resolved [47].

**Figure 11:**
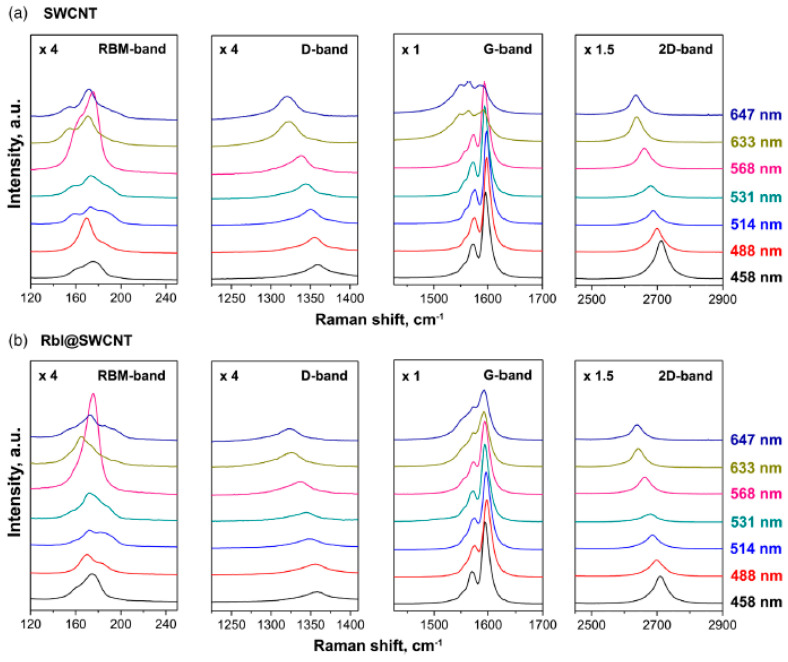
Raman spectra of (**a**) pristine SWCNTs and (**b**) rubidium-iodide-filled SWCNTs acquired at laser wavelengths of 458, 488, 514, 531, 568, 633, 647 nm. Reprinted with permission from Ref. [47], copyright 2019 Wiley-VCH Verlag GmbH & Co. KGaA, Weinheim.

Raman spectroscopy investigations of pristine and rubidium-iodide-filled SWCNTs acquired at laser wavelengths of 458, 488, 514, 531, 568, 633, and 647 are shown in Figure 11 [47]. This example demonstrates n-doping and there are similar changes. Underlying mechanism of p and n-doping changes, and there are different shifts, modification of profiles, intensities, and number of peaks. 

**Figure 12:**
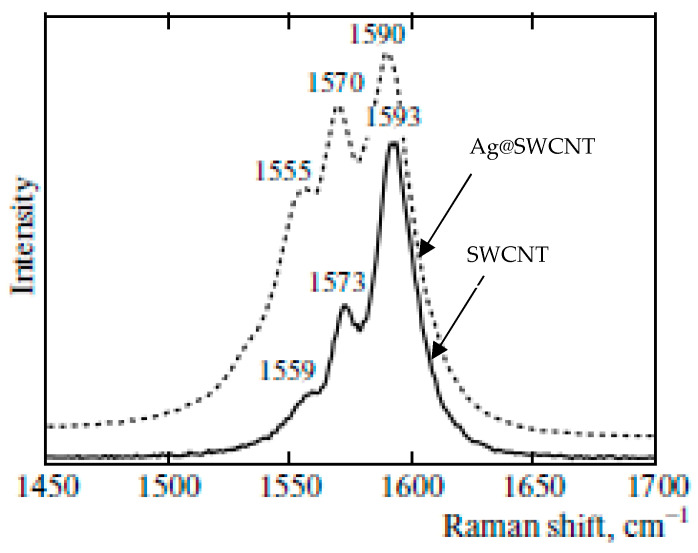
G-band of the pristine (solid line) and silver-filled (dashed line) SWCNTs. The numbers show the position of the peaks. Reproduced from M. V. Kharlamova et al. Donor doping of single-walled carbon nanotubes by filling of channels with silver, Journal of Experimental and Theoretical Physics, V. 115, № 3, p. 485–491 [55]. copyright 2012, Springer Nature.

Figure 12 shows the G-band of the pristine and silver-filled SWCNTs [55]. The spectra show the increase in intensity of G_BWF_ and G_TO_ components in comparison to the G_LO_ component, which corresponds to n-doping. The underlying mechanism is discussed in Ref. [47]. 

**Figure 13:**
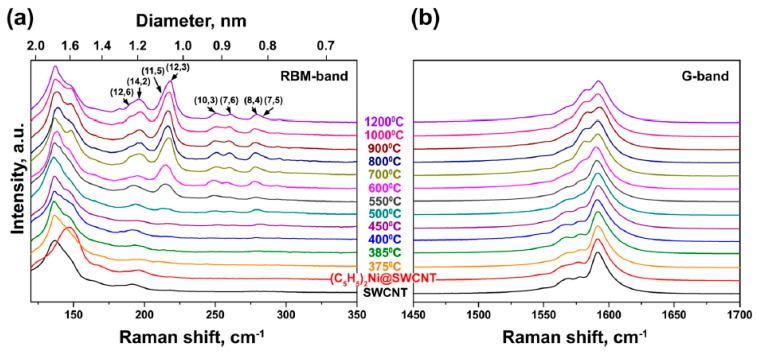
The RBM (**a**) and G-bands (**b**) of Raman spectra of the pristine, nickelocene-filled SWCNTs and samples annealed at 375 to 1200 °C acquired at laser wavelengths of 647 nm. The chiralities of tubes are denoted. Reprinted with permission from Ref. [63], copyright 2015 Wiley-VCH Verlag GmbH & Co. KGaA, Weinheim.

Figure 13 shows the RBM and G-bands of Raman spectra of the pristine, nickelocene-filled SWCNTs and samples annealed at 375 to 1200 °C [63]. The peaks of inner tubes appear in the spectra during annealing. Changes in the electronic properties are not recognized. 

**Figure 14:**
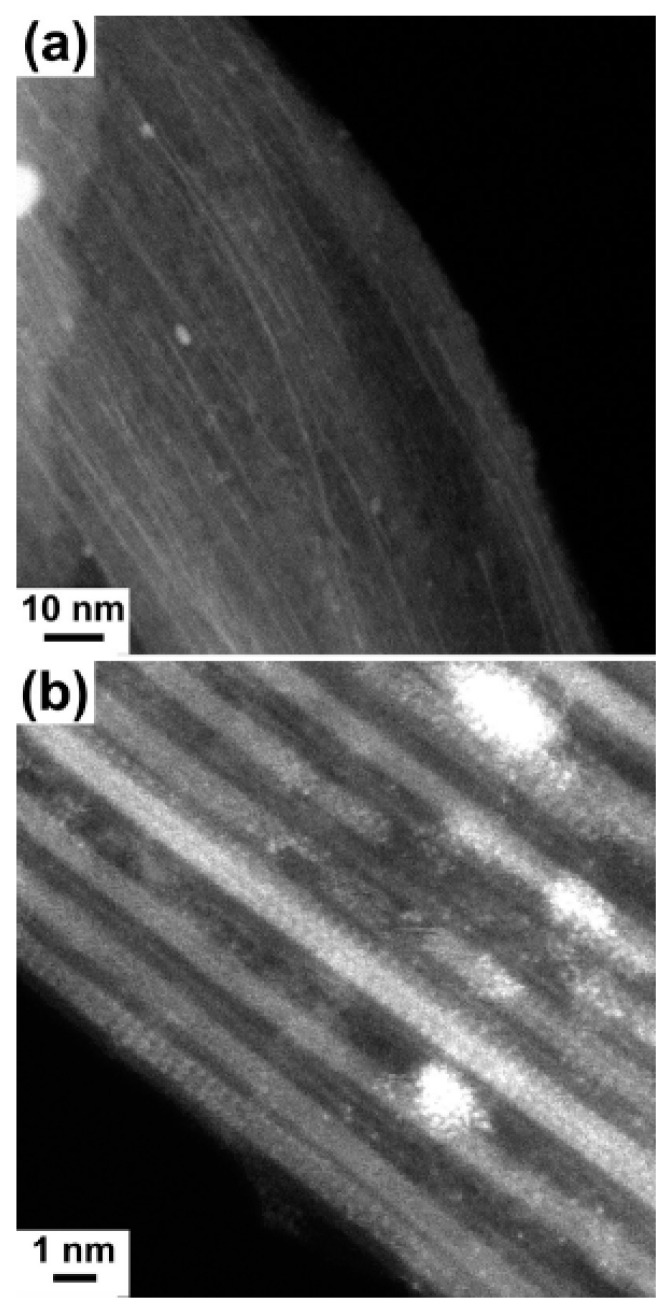
The silver-chloride-filled SWCNTs. (**a**) The low-magnification image of filled SWCNTs. (**b**) The high-magnification image of filled SWCNTs. Reprinted with permission from Ref. [39], copyright 2015 Wiley-VCH Verlag GmbH & Co. KGaA, Weinheim.

The diameter-dependence of Raman spectroscopy spectra of silver chloride-filled SWCNTs was studied in Ref. [39]. Figure 14 shows the HR TEM data of the silver-chloride-filled SWCNTs [39]. The data show the filled nanotube channels. The filler material is visible because the nanotube channels are filled. 

**Figure 15:**
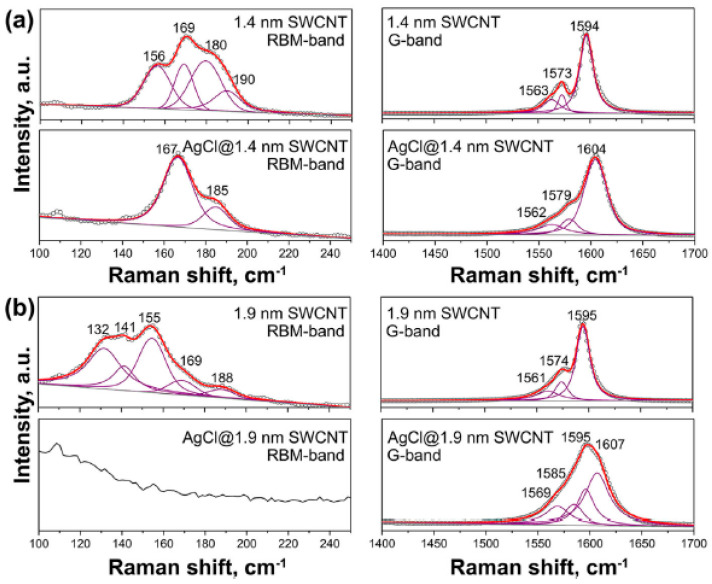
The RBM and G-band of the pristine and silver chloride-filled SWCNTs with diameter of (**a**) 1.4 nm and 1.9 nm (**b**). Reprinted with permission from Ref. [39], copyright 2015 Wiley-VCH Verlag GmbH & Co. KGaA, Weinheim.

Figure 15 shows Raman spectra of 1.4 nm- and 1.9 nm-diameter SWCNTs filled with silver chloride fitted with individual components [39]. The RBM-band of Raman spectra of filled SWCNTs is fitted with components of different-diameter SWCNTs. The G-band of filled SWCNTs is fitted with the components of tangential and longitudinal phonons for the pristine and filled nanotubes. It is shown that the RBM-band of the large 1.9 nm-diameter SWCNTs disappears upon fillings; this is also reflected in the G-band. 

**Figure 16:**
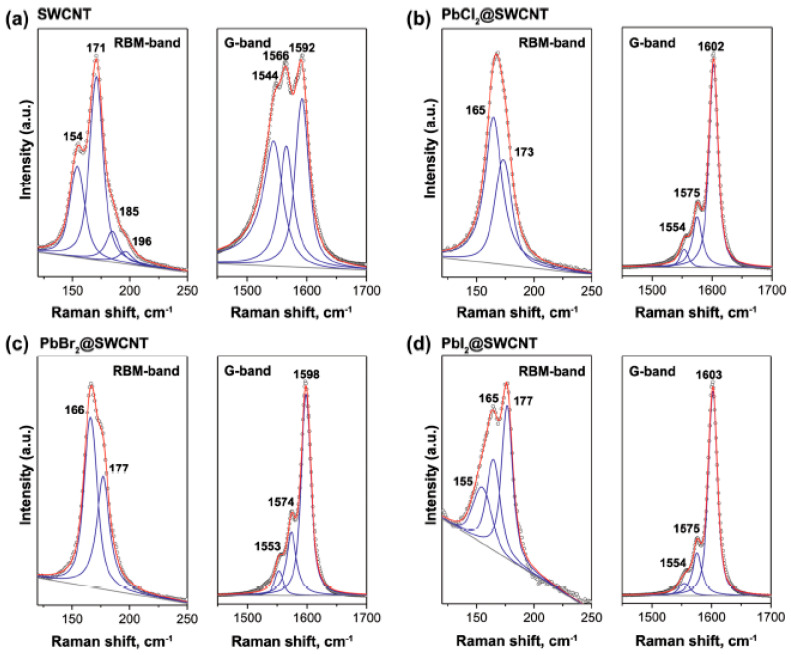
The RBM and G-bands of Raman spectra of the pristine (**a**) and lead-chloride- (**b**), lead-bromide- (**c**), and lead-iodide-filled SWCNTs (**d**). The numbers denote the positions of peaks. Reproduced from M. V. Kharlamova et al. Revealing the doping effect of encapsulated lead halogenides on single-walled carbon nanotubes, Applied Physics A, V. 125, article number 320, 2019, Springer Nature [46].

**Figure 17:**
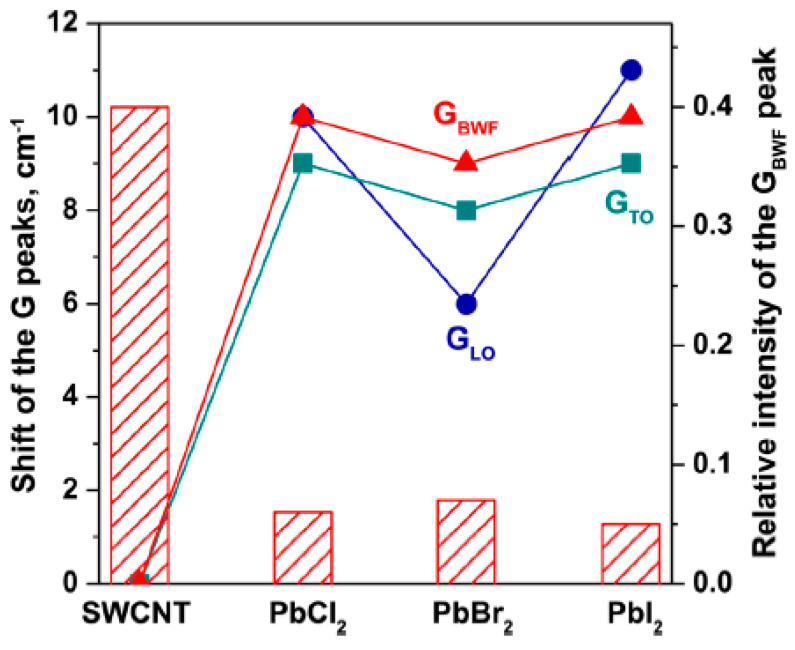
The shift in the G-band peaks (G_BWF_, G_TO_, G_LO_) and relative intensity of the G_BWF_ peak for the pristine SWCNTs, and lead-chloride-, lead-bromide-, and lead iodide-filled SWCNTs. Reproduced from M. V. Kharlamova et al. Revealing the doping effect of encapsulated lead halogenides on single-walled carbon nanotubes, Applied Physics A, V. 125, article number 320, 2019, Springer Nature [46].

Ref. [46] presents Raman spectroscopy study of pristine and lead halogenides-filled SWCNTs. Figure 16 shows Raman spectra of the pristine and filled SWCNTs fitted with individual components acquired at lase wavelengths of 633 nm. The Raman spectra show modifications of peak positions, peak shapes, and profiles in the filled SWCNTs. The shifts and relative intensities of G_BWF_, G_LO_, and G_TO_ components differ for different salts inside SWCNTs (Figure 17) [46]. 

Thus, the application of Raman spectroscopy allows for the investigation of the electronic properties of filled SWCNTs. The changes in spectra vary for p- and n-doping. They also vary for different-diameter SWCNTs. 

## 8. Near Edge X-Ray Absorption Fine Structure Spectroscopy

**Figure 18:**
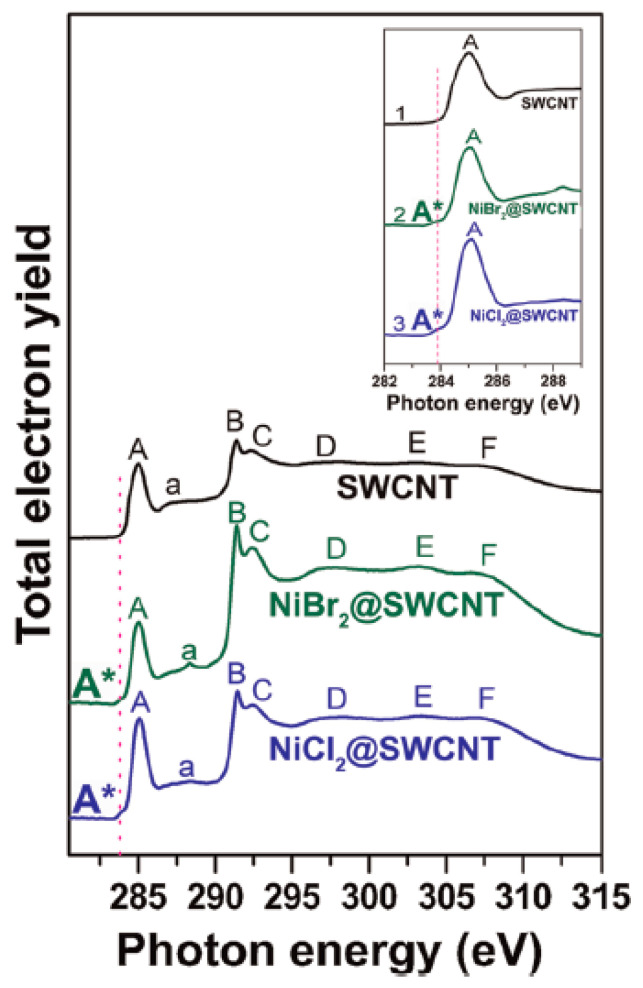
The NEXAFS spectra of the pristine SWCNTs and nanotubes filled with nickel chloride and nickel bromide. The inset shows the new peak A*. Reprinted with permission from Ref. [27], copyright 2015 Wiley-VCH Verlag GmbH & Co. KGaA, Weinheim.

The local bonding in filled SWCNTs was studied by NEXAFS. The spectrum of the pristine SWCNTs includes the peaks A, a, B, C, D, E, F that correspond to the fine structure of the band edge (Figure 18) [27]. The new peak A* appears in the spectra upon filling that corresponds to the formation of new bonds between *π*^∗^-orbitals of introduced compounds and carbon nanotube walls. 

Thus, NEXAFS allows investigating of bond environment in filled SWCNTs, and reveal the differences in chemical bonds before and after filling with different substances. 

## 9. Photoemission Spectroscopy

**Figure 19:**
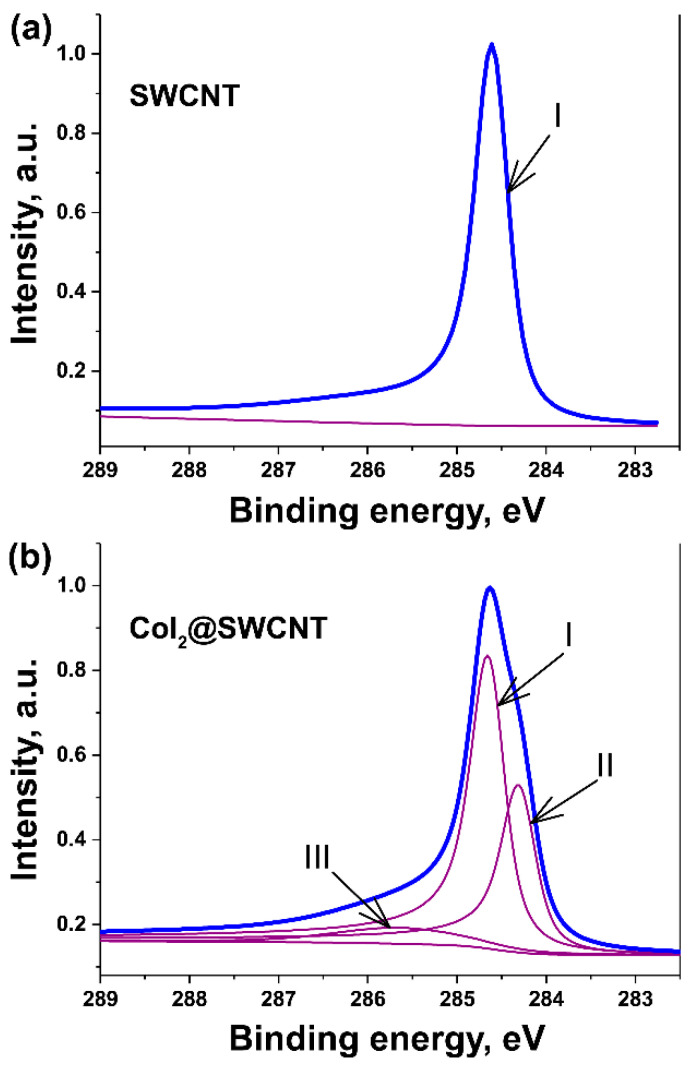
The C 1s XPS spectra of the pristine (**a**) and cobalt-iodide-filled SWCNTs (**b**) fitted with individual components.

The electronic properties were also investigated by XPS. Figure 19 shows the C 1s XPS spectra of the pristine and cobalt-iodide-filled SWCNTs fitted with individual components. The spectrum of the pristine SWCNTs is fitted with one component. The spectrum of the filled SWCNTs is fitted with three components. The first component corresponds to unfilled SWCNTs. The second component belongs to the filled SWCNTs, and it is shifted by about 0.3 eV to low energy region, which corresponds to p-doping of SWCNTs. The third component is under debate. 

**Figure 20:**
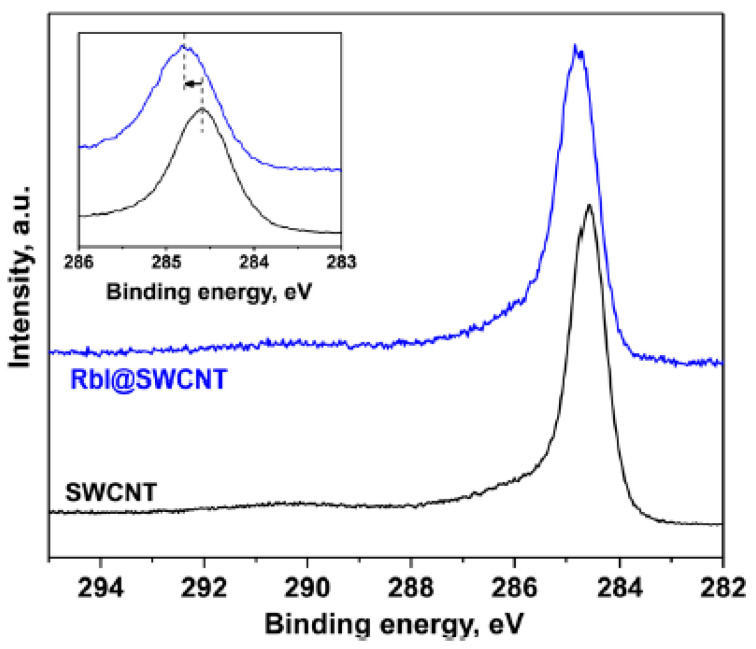
The C 1s XPS spectra of the pristine and rubidium-iodide-filled SWCNTs. The insert shows the shift in peak. Reprinted with permission from Ref. [47], copyright 2015 Wiley-VCH Verlag GmbH & Co. KGaA, Weinheim.

Figure 20 demonstrates the C 1s XPS spectra of pristine SWCNTs and nanotubes filled with electron donor, rubidium iodide, which leads to n-doping of SWCNTs [47]. The spectrum of the filled SWCNTs is shifts by 0.2 eV to the high-energy region.

**Figure 21:**
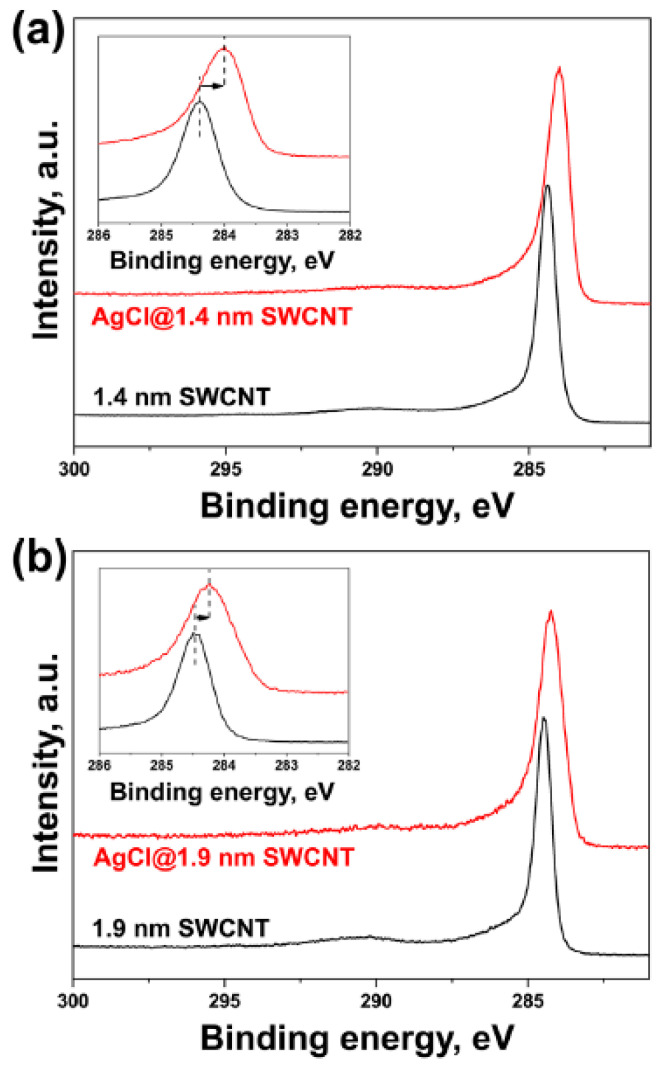
The C 1s XPS spectra of pristine and silver-chloride-filled SWCNTs with diameter of 1.4 nm (**a**) and 1.9 nm (**b**). The insert shows the shift in peak. Reprinted with permission from Ref. [39], copyright 2015 Wiley-VCH Verlag GmbH & Co. KGaA, Weinheim.

Figure 21 shows the C 1s XPS spectra of pristine and silver chloride filled SWCNTs with diameter of 1.4 nm and 1.9 nm [39]. The peak of filled 1.4 nm-diameter SWCNTs is shifted by about 0.4 nm, and the peak of filled 1.9 nm diameter SWCNTs is shifted to about 0.2 eV to the high-energy region. This corresponds to the p-doping of SWCNTs produced by arc-discharge and chemical vapor deposition methods. 

Thus, PES allows for investigation of the effects of filler on SWCNTs. This is performed for n- and p-type dopants, and the changes in the spectra are opposite. This also allows revealing modifications in the spectra of different-diameter SWCNTs. 

## 10. Optical Absorption Spectroscopy

**Figure 22:**
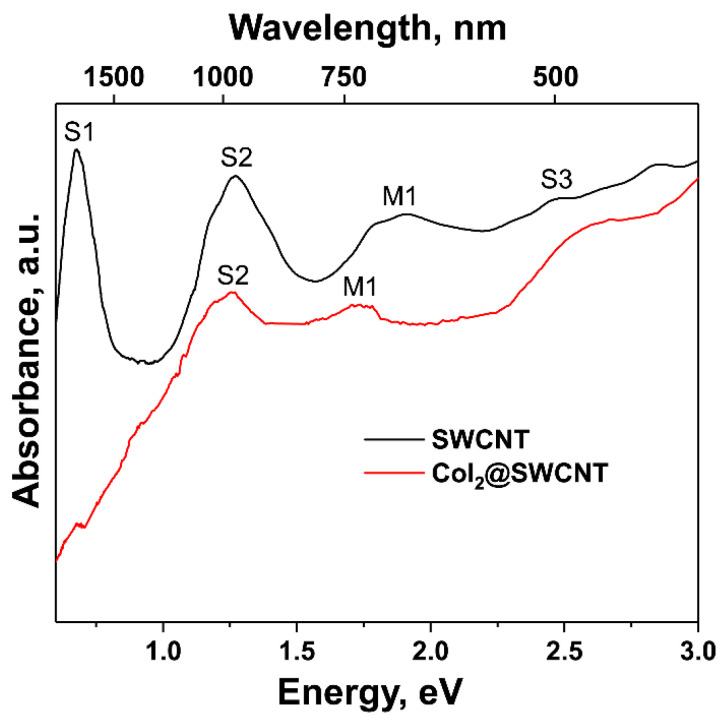
The OAS spectra of the pristine and cobalt-iodide-filled SWCNTs.

The optical absorption spectroscopy was also applied to investigate the electronic properties of pristine and filled SWCNTs. The OAS spectra of the pristine and cobalt-iodide-filled SWCNTs are shown in Figure 22. The peaks of electronic transitions between conduction and valence band are denoted. For the cobalt-iodide-filled SWCNTs, the peaks disappear, their intensity decreases, they shift and change shape. This corresponds to the doping of SWCNTs by introduced compound. 

**Figure 23:**
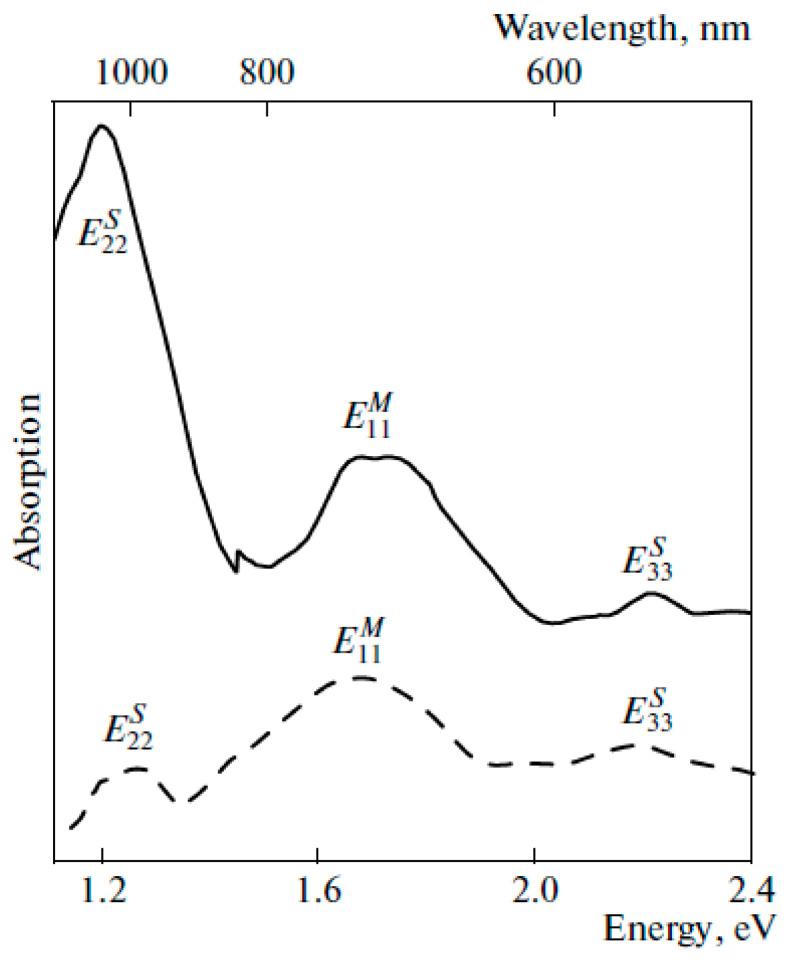
The OAS spectra of the pristine and silver-filled SWCNTs. Reproduced from M. V. Kharlamova et al. Donor doping of single-walled carbon nanotubes by filling of channels with silver, Journal of Experimental and Theoretical Physics, V. 115, № 3, p. 485–491, 2012, Springer Nature [55].

Figure 23 shows the OAS spectra of pristine SWCNTs and nanotubes filled with the electron donor, silver [55]. The spectra demonstrate the shift in peaks, decrease in peak intensity, and change in peak shape in the case of filled SWCNTs. 

Thus, OAS allows finding the modification in spectra of filled SWCNTs that are dependent on doping of SWCNTs with n- and p-dopants. The changes can be different, and also depend on the diameter of the SWCNTs.

## 11. Conclusions: Challenges and Future

There are several limitations:

At the moment, the adoption of applications of filled SWCNTs is bottlenecked by their lab-scale synthesis. The supply situation is even more dramatic for metallicity sorted SWCNT for filling as two different lab-scale techniques have to be combined. Metallicity sorting is currently still challenging in regard to yield, purity, and also the shortened lengths of metallicity sorted or single-chirality SWCNTs. There are different techniques actively developed and improved. However, there are currently none that could sustain commercially viable applications. The demand for raw material dictates the development of controlled scalable low-cost methods for synthesis, purification and separation of SWCNTs. The processes for filling SWCNTs with different substances, have so far only been demonstrated at the lab scale. Applications need to be able to rely on consistent high and uniform yields of filled SWCNT. These quality standards have to be maintained when up-scaling the production to industrial levels. If and how fast these challenges can be overcome will greatly depend on how well the scientific community can built up an understanding of synthesis, purification, sorting, and filling mechanisms of SWCNTs. This does also comprise investigating in detail the process parameters for the best filling method for each substance. While the application potential of SWCNTs and filled SWCNTs has been explored in applied research, still more basic research is likely needed before the ultimate goal of commercial availability can become a reality.

There is a number of open challenges for applications of filled SWCNTs, but considering the recent advancements in purification, synthesis and filling of SWCNTs rapid progress, that will overcome many of these challenges, can be expected in the near future [105,106]. These achievements will doubtlessly inspire visions for many more potential applications in this emerging research area.

Future perspectives include the following:

The unique combination of exceptional material properties of SWCNTs is promising for a wide range of applications. A very specific feature of SWCNTs is that their electronic properties crucially depend on their diameter and twist angle, the chirality. So many potential applications hinge on the effective control of the electronic properties. In the past years, considerable progress has been made in controlling the electronic properties of SWCNTs by filling them with appropriate materials. Further progress is expected in controlled electronic properties of filled SWCNTs, which will, in conjunction with the wide range of demonstrated and envisaged applications, stimulate further advancements towards viable technologies built on them.

The synthesis and separation of metallic and semiconducting or even single chiralities of SWCNTs vastly extends the scope of possible applications for filled SWCNT. This is true especially for applications that rely on specific electronic properties. If there is more control on electronic properties of the starting material, then there is also more control on the achievable results if the electronic properties are modified by filling SWCNT. This new level of attainable accuracy in engineering electronic properties by choosing a type or even single chirality of SWCNT as well as an appropriate dopant for filling will greatly benefit most of the applications. 

The electronic properties of cobalt-iodide- and nickel-filled carbon nanotubes can be applied in various fields, which has been the basis of work for hundreds of years in all countries. It became a trend to start papers by mentioning early Soviet papers; this will continue as more and more immigrant scientists become Noble prize winners. I think that this topic is not interesting as the investigation of metals; however, the study of metal-filled single-walled carbon nanotubes produced by different methods, including treatments of metallocene-filled SWCNTs and the resulting metals, is an appealing topic for future research around the world. 

## Figures and Tables

**Table 1:** The fitting of RBM and G-band of Raman spectra of pristine and cobalt diiodide-filled SWCNTs with individual components (C1, C2, G_BWF_, G_TO_, G_LO_) obtained at laser wavelengths of 633 nm and 785 nm.

Sample	RBM, cm^−1^		G-Band, cm^−1^		
	C1	C2	G_BWF_	G_TO_	G_LO_
λ_ex_ = 633 nm					
SWCNT	151	159	1546	1565	1591
CoI_2_@SWCNT	162	172	1557	1576	1603
λ_ex_ = 785 nm					
SWCNT	157	171	1556	1571	1593
CoI_2_@SWCNT	159	172	1560	1577	1604

## Data Availability

Data are available on request to the first author (Marianna V. Kharlamova) of every reviewed paper.
